# Understanding the use intention and influencing factors of telerehabilitation in people with rehabilitation needs: a cross-sectional survey

**DOI:** 10.3389/fpubh.2023.1274080

**Published:** 2023-10-31

**Authors:** Mao-Yuan Wang, Hong Chen, Cheng Gong, Xu-Miao Peng, Yan-Biao Zhong, Chun-Mei Wu, Yun Luo, Yong-Qiang Wu

**Affiliations:** ^1^Department of Rehabilitation Medicine, The First Affiliated Hospital of Gannan Medical University, Ganzhou, Jiangxi Province, China; ^2^Ganzhou Key Laboratory of Rehabilitation Medicine, Ganzhou, Jiangxi Province, China; ^3^Ganzhou Intelligent Rehabilitation Technology Innovation Center, Ganzhou, Jiangxi Province, China; ^4^Shaanxi Rehabilitation Hospital, Xi'an, Shaanxi Province, China; ^5^School of Public Health and Health Management, Gannan Medical University, Ganzhou, Jiangxi Province, China; ^6^Department of Rehabilitation Medicine, Xi’an Children’s Hospital, Xi’an, Shaanxi Province, China

**Keywords:** telerehabilitation, UTAUT, use intention, influencing factors, rehabilitation needs

## Abstract

**Objective:**

This study aimed to investigate the use intention and influencing factors of telerehabilitation in people with rehabilitation needs.

**Methods:**

This cross-sectional survey recruited a total of 183 participants with rehabilitation needs from May 2022 to December 2022. Sociodemographic and medical data were collected by a structured questionnaire. The factors influencing the use intention of telerehabilitation were measured by the extended Unified Theory of Acceptance and Use of Technology (UTAUT) model. Multiple hierarchical regression analyses were performed.

**Results:**

A total of 150 valid questionnaires were included for analysis. The results indicated that the use intention of telerehabilitation was overall high in people with rehabilitation needs. Health condition (*β* = −0.21, *p* = 0.03), performance expectancy (*β* = 0.21, *p* = 0.01), facilitating conditions (*β* = 0.25, *p* = 0.03), perceived trust (*β* = 0.25, *p* < 0.01), and self-efficacy (*β* = 0.19, *p* = 0.04) were significant factors influencing the use intention of telerehabilitation.

**Conclusion:**

Overall, the use intention of telerehabilitation is high in individuals with rehabilitation needs. Health conditions, performance expectancy, facilitating conditions, perceived trust, and self-efficacy are important factors influencing the use intention of telerehabilitation in individuals with rehabilitation needs.

## Introduction

With the aging of the population and the rising incidence of disabling diseases, the number of people with rehabilitation needs is rapidly growing worldwide (1, 2). These people with severe functional impairment often require long-term and persistent rehabilitation services to improve their functional limitations and quality of life, which results in a huge health and economic burden on their families and society (3, 4). The World Health Organization indicates that the globe is facing challenges related to the increasing unmet rehabilitation needs in this century, especially in some low-income and middle-income countries (5). According to a survey, the rehabilitation needs of Chinese older adult have increased more than 70% in the past 30 years, which is much higher than the world average (6).

In order to redress the imbalance between the supply and demand of rehabilitation services, some healthcare organizations or countries attempted to use telerehabilitation to provide professional rehabilitation services for people with rehabilitation needs, including consultation, education, assessment, monitoring, and treatment (7–10). However, telerehabilitation is currently still in the early stages of implementation in most areas (11). The use intention and influencing factors of telerehabilitation remain unclear in people with rehabilitation needs. Therefore, a comprehensive understanding of the use intention and influencing factors of telerehabilitation is a prerequisite to facilitate its application.

The Unified Theory of Acceptance and Use of Technology (UTATU) model is one of the most widely used models for exploring behavioral intention (BI) to use new technologies, and it explains approximately 70% of the variance in behavioral intentions (12). The UTAUT model includes four core predictors: Performance expectancy (PE), effort expectancy (EE), social influence (SI), and facilitating conditions (FC) (13). Currently, several studies have applied the UTAUT model in the context of telemedicine to analyze the underlying factors influencing users’ behavioral intentions to adopt telemedicine (14–16). However, some studies suggest that the variables of the UTAUT model cannot fully explain users’ BI to telemedicine (17). Therefore, some studies have extended the original UTAUT model by integrating context-specific determinants to improve its predictive power. For example, Zhu et al. (14), Breil et al. (18), and Li et al. (19) extended the UTAUT model by applying perceived risk (PR), perceived trust (PT), and self-efficacy (SE) and concluded that these are also important factors that influence users’ use of telemedicine. In addition, a recent review indicated that patient characteristics (e.g., age, gender, educational level, and occupation) and health conditions were also important factors influencing patient’s use of telemedicine (20).

Therefore, this study aimed to investigate the use intention and influencing factors of telerehabilitation in people with rehabilitation needs in terms of three aspects: patients’ sociodemographic characteristics, medical characteristics, and an extended UTAUT theoretical model.

## Methods

### Study design and participants

A cross-sectional survey was conducted to investigate the behavioral intentions to use telerehabilitation among people with rehabilitation needs. Participants were recruited in the Department of Rehabilitation Medicine, First Affiliated Hospital of Gannan Medical University from May 2022 to December 2022. The department covers a wide range of the most common rehabilitation populations: cerebrovascular diseases, musculoskeletal chronic pain, post-operative fracture, etc.

The inclusion criteria of participants in this study were patients aged 14 years or older, who had completed at least 1 week of rehabilitation therapy. This questionnaire was completed on-site by the participants or their direct relatives in a paper form. Before the start of the survey, the researchers introduced the background, content, and objectives of this survey to each participant to ensure their informed consent, and this questionnaire was anonymous and voluntary. This study was approved by the Ethics Committee of the First Affiliated Hospital of Gannan Medical University.

### Measures

The first section of this survey questionnaire used a structured questionnaire to collect the sociodemographic data (e.g., gender, age, occupation, and educational level) and medical data (e.g., diagnosis, impact of health condition on one’s life, and telerehabilitation experience) of the participants. The second section was to identify the factors influencing users’ behavioral intention to use telerehabilitation using an extended UTAUT model. The 28-item extended UTAUT questionnaire consists of eight constructs that influence behavioral intention to use telerehabilitation: PE, EE, SI, FC, PR, PT, SE, and BI. We defined each variables based on the specific context of telerehabilitation: PE was defined as the extent to which individuals believe that using the telerehabilitation will help them to improve in functional performance; EE was defined as the degree of ease associated with the use of the telerehabilitation; SI was defined as the extent to which individuals are impacted by the opinions of surrounding groups; FC was defined as the extent to which an individual believes that an organizational and technical infrastructure exists to support the use of the telerehabilitation; PR was defined as an individual’s expectation of the impact of uncertainty or loss resulting from the use of telerehabilitation; PT was defined as the extent to which individual perceives telerehabilitation to be reliable and trustworthy; SE was defined as the extent to which individual perceives that he or she can successfully use telerehabilitation; BI was defined as the extent to which individuals tendency to use or recommend an telerehabilitation. All items are measured with a 5-point Likert scale, ranging from (1) “strongly disagree” to (5) “strongly agree.”

To ensure the reliability and validity of this questionnaire, all items were adopted or modified from previous studies. The test results showed the Cronbach’s alpha for each construct ranged from 0.754 to 0.882, the average variance extracted (AVE) ranged from 0.619 to 0.765, and the square root of AVE for each construct was greater than the correlation coefficient between other constructs and itself, indicating good reliability and validity (17). This survey questionnaire is detailed in Appendix 1.

### Statistical analysis

SPSS 25.0 (SPSS Inc., Chicago, IL, United States) was used for descriptive, univariate, and multivariate statistical analysis. Between-group differences in sociodemographic and medical characteristics at BI were analyzed using analysis of variance and independent t-tests. Correlations for the eight constructs of the extended UTAUT were analyzed using Pearson correlation coefficients. Multiple hierarchical regression analysis was performed to investigate the possible predictors of the adoption intention of telerehabilitation. With all regression assumptions satisfied, BI was considered as the dependent variable and the independent variables were sequentially entered into the regression model in three modules: (1) sociodemographic data, (2) medical data, and (3) extended UTAUT predictors. A two-tailed value of *p* < 0.05 was considered as statistically significant.

We used Smart-PLS 4.0 (free trial version) to test the reliability and validity. A Cronbach’s alpha higher than 0.7 indicates higher internal consistency. The average variance extracted (AVE) higher than 0.5 and the square root of AVE for each construct greater than the correlation coefficient between other constructs and itself indicate a good convergent validity (17).

## Results

### Sociodemographic and medical characteristics

A total of 183 questionnaires were collected, including 150 valid questionnaires and 33 invalid questionnaires (15 were completed in less than 1 min, 13 had the same obvious answer, 5 had unfilled options in the questionnaire), with a validity rate of 81.97%. The sociodemographic and medical characteristics of 150 participants are shown in Table 1. The majority of participants stated that they had not previously used telerehabilitation, and only 29 (19.3%) had previously used telerehabilitation.

**Table 1:** Demographic sample characteristics.

Variables		*n*	Percentage (%)	Behavioral intention [Mean (SD)]	*P*
Gender	Male	79	52.7	4.10 (0.59)	0.65
	Female	71	47.3	4.06 (0.48)	
Age (years)	≤ 30	37	24.7	4.03 (0.47)	0.82
	31–40	40	26.7	4.14 (0.73)	
	41–50	30	20.0	4.12 (0.44)	
	51–60	21	14.0	4.00 (0.41)	
	≥60	22	14.7	4.09 (0.47)	
Educational level	Primary school and below	18	12.0	3.98 (0.43)	0.08
	Middle school	43	28.7	3.95 (0.67)	
	Senior high\secondary Schools	27	18.0	4.01 (0.38)	
	Undergraduate\junior college	55	36.7	4.23 (0.51)	
	Master’s and above	7	4.7	0.42 (0.16)	
Occupation	Party and government agencies\institutional workers	33	22.0	4.29 (0.50)	0.14
	Enterprise staff	27	18.0	3.99 (0.61)	
	Self-employed\freelance	29	19.3	4.07 (0.65)	
	Physical laborers	24	16.0	3.92 (0.42)	
	Students	7	4.7	4.14 (0.38)	
	Retired\unemployed\non-working	30	20.0	4.07 (0.47)	
Monthly family income/capita (RMB)	<2000	22	14.7	4.05 (0.58)	0.20
	2000–3,000	12	8.0	3.97 (0.50)	
	3,001–4,000	36	24.0	4.08 (0.47)	
	4,001–5,000	31	20.7	3.94 (0.53)	
	>5,000	49	32.7	4.22 (0.57)	
Impact of health condition on one’s life	No effect	10	6.7	3.73 (0.41)	0.18
	Mild impact	47	31.3	4.11 (0.49)	
	Moderate impact	41	27.3	4.15 (0.55)	
	Severe impact	52	34.7	4.08 (0.58)	
Forms of medical costs	Self-funded	36	24.0	4.06 (0.54)	0.46
	Employee Basic Medical Insurance	56	37.3	4.15 (0.54)	
	Basic medical insurance for urban and rural residents	42	28.0	3.98 (0.51)	
	Publicly funded medical care	5	3.3	4.27 (0.72)	
	Commercial Insurance	4	2.7	3.83 (0.58)	
	Other	7	4.7	4.24 (0.53)	
Diagnostic groups	Cerebrovascular diseases	42	28.0	4.04 (0.53)	0.16
	Musculoskeletal chronic pain	50	33.3	4.22 (0.58)	
	Post-operative fracture	24	16.0	4.00 (0.61)	
	Other	34	22.7	3.99 (0.40)	
Telerehabilitation experience	Used	29	19.3	4.18 (0.48)	0.26
	Not used	121	80.7	4.06 (0.55)	
Overall behavioral intention				4.08 (0.54)	

### Adoption of telerehabilitation

The use intention of telerehabilitation was overall high in patients with rehabilitation needs with a mean of 4.08 (SD 0.54; Table 1) (21). There were no statistical differences in BI for all groups of sociodemographic and medical characteristics (Table 1; *p* > 0.05).

### Correlation between constructs

Table 2 indicates that PE, EE, SI, FC, PR, PT, and SE had a significant positive correlation with BI. However, no significant correlation was found between BI and PR.

**Table 2:** Correlation analysis.

	PE	EE	SI	FC	PR	PT	SE	BI
PE	1							
EE	0.609**	1						
SI	0.591**	0.526**	1					
FC	0.581**	0.756**	0.626**	1				
PR	−0.074	−0.138	−0.034	−0.074	1			
PT	0.598**	0.538**	0.491**	0.602**	−0.180*	1		
SE	0.547**	0.687**	0.509**	0.692**	−0.15	0.626**	1	
BI	0.637**	0.566**	0.570**	0.695**	−0.045	0.657**	0.628**	1

*p < 0.05; **p < 0.01.

### Multiple hierarchical regression analysis

Table 3 summarizes the results of multiple hierarchical regression analysis. Sociodemographic data were included in the first step (*R^2^* = 0.110, ∆*R^2^* = 0.01, *F* = 1.10, *p* = 0.36) and explained 11% of the variance of BI. Sociodemographic characteristics were not significant factors influencing BI for telerehabilitation.

**Table 3:** Hierarchical regression model of intention.

Predictors	B	*β*	*t*	*R^2^*	∆*R^2^*	*P*	VIF
Step 1: Sociodemographic data				0.110	0.010		
*Constant*	3.985		12.336			0.000	
Age	0.002	0.071	0.631			0.529	
Gender: male	0.065	0.061	0.688			0.493	
Educational level: Primary school and below	−0.094	−0.057	−0.326			0.745	
Educational level: middle school	−0.110	−0.093	−0.427			0.670	
Educational level: senior high\secondary schools	−0.051	−0.037	−0.200			0.842	
Educational level: Undergraduate\junior college	0.107	0.096	0.474			0.636	
Occupation: party and government agencies\institutional workers	0.175	0.135	0.933			0.352	
Occupation: enterprise staff	−0.029	−0.021	−0.159			0.874	
Occupation: self-employed\freelance	0.034	0.025	0.192			0.848	
Occupation: physical laborers	−0.124	−0.085	−0.767			0.444	
Students	0.087	0.034	0.317			0.752	
Income: <2000	0.009	0.006	0.053			0.958	
Income: 2000–3,000	−0.037	−0.019	−0.181			0.857	
Income: 3001–4,000	−0.015	−0.012	−0.106			0.916	
Income: 4001–5,000	−0.226	−0.170	−1.664			0.099	
Step 2: Medical data				0.183	0.002		
*Constant*	4.282		10.486			0.000	
Age	0.001	0.035	0.265			0.791	
Gender: male	0.023	0.021	0.227			0.821	
Educational level: Primary school and below	−0.020	−0.012	−0.063			0.950	
Educational level: middle school	−0.065	−0.055	−0.234			0.815	
Educational level: senior high\secondary schools	0.012	0.009	0.044			0.965	
Educational level: undergraduate\junior college	0.155	0.140	0.660			0.510	
Occupation: party and government agencies\institutional workers	0.085	0.066	0.411			0.682	
Occupation: enterprise staff	−0.099	−0.071	−0.509			0.612	
Occupation: self-employed\freelance	−0.004	−0.003	−0.021			0.983	
Occupation: physical laborers	−0.154	−0.105	−0.900			0.370	
Occupation: students	0.014	0.005	0.046			0.964	
Income: <2000	−0.004	−0.003	−0.021			0.983	
Income: 2000–3,000	−0.121	−0.061	−0.561			0.576	
Income: 3001–4,000	−0.036	−0.028	−0.237			0.813	
Income: 4001–5,000	−0.226	−0.171	−1.545			0.125	
Health condition: no effect	−0.449	−0.209	−2.153			0.033	
Health condition: mild impact	−0.046	−0.040	−0.379			0.705	
Health condition: moderate impact	−0.031	−0.026	−0.253			0.800	
Forms: self-funded	−0.215	−0.172	−0.900			0.370	
Forms: employee basic medical insurance	−0.243	−0.220	−1.001			0.319	
Forms: basic medical insurance for urban and rural residents	−0.293	−0.245	−1.246			0.215	
Forms: Publicly funded medical care	0.092	0.031	0.264			0.793	
Forms: commercial insurance	−0.321	−0.097	−0.872			0.385	
Telerehabilitation experience: used	0.114	0.084	0.933			0.352	
Diagnostic: cerebrovascular diseases	0.041	0.034	0.264			0.792	
Diagnostic: musculoskeletal chronic pain	0.134	0.118	1.031			0.305	
Diagnostic: post-operative fracture	−0.046	−0.031	−0.286			0.775	
Step 3: UTAUT				0.681	0.586		
*Constant*	0.853		2.063			0.041	
Age	0.001	0.027	0.301			0.764	
Gender: male	−0.007	−0.007	−0.106			0.916	
Educational level: primary school and below	−0.155	−0.094	−0.755			0.452	
Educational level: middle school	−0.201	−0.170	−1.080			0.283	
Educational level: senior high\secondary schools	−0.058	−0.042	−0.318			0.751	
Educational level: undergraduate\junior college	0.009	0.008	0.057			0.954	
Occupation: party and government agencies\institutional workers	−0.023	−0.018	−0.170			0.866	
Occupation: enterprise staff	−0.093	−0.066	−0.722			0.472	
Occupation: self-employed\freelance	−0.147	−0.108	−1.166			0.246	
Occupation: physical laborers	−0.001	−0.001	−0.010			0.992	
Students	0.111	0.044	0.561			0.576	
Income: <2000	−0.036	−0.024	−0.293			0.770	
Income: 2000–3,000	−0.029	−0.015	−0.207			0.837	
Income: 3001–4,000	0.004	0.003	0.039			0.969	
Income: 4001–5,000	−0.093	−0.070	−0.949			0.344	
Health condition: no effect	−0.068	−0.032	−0.477			0.634	
Health condition: mild impact	−0.015	−0.013	−0.192			0.848	
Health condition: moderate impact	0.013	0.011	0.160			0.873	
Forms: self-funded	−0.251	−0.200	−1.550			0.124	
Forms: employee basic medical insurance	−0.340	−0.307	−2.122			0.036	
Forms: basic medical insurance for urban and rural residents	−0.273	−0.228	−1.752			0.082	
Forms: publicly funded medical care	−0.079	−0.026	−0.338			0.736	
Forms: commercial insurance	−0.247	−0.074	−1.037			0.302	
Telerehabilitation experience: used	−0.066	−0.049	−0.804			0.423	
Diagnostic: cerebrovascular diseases	0.182	0.153	1.690			0.094	
Diagnostic: musculoskeletal chronic pain	0.076	0.067	0.866			0.389	
Diagnostic: post-operative fracture	0.096	0.066	0.901			0.370	
PE	0.186	0.214	2.583			0.011	2.128
EE	−0.050	−0.059	−0.551			0.583	2.869
SI	0.097	0.111	1.369			0.174	1.897
FC	0.230	0.252	2.259			0.026	3.200
PR	0.044	0.059	0.963			0.337	1.055
PT	0.226	0.249	3.040			0.003	2.068
SE	0.182	0.192	2.093			0.039	2.470

The second step included medical data as predictors (*R^2^* = 0.183, ∆*R^2^* = 0.002, *F* = 1.01, *p* = 0.46), and explained 18.3% of the variance of BI. In this step, a significant factor influencing BI was health condition: no effect (*β* = −0.21, *p* = 0.03).

The extended UTAUT predictors were included in the third step of hierarchical regression analysis (*R^2^* = 0.68, ∆*R^2^* = 0.59, *F* = 7.21, *p* < 0.001) and explained 68% of the variance of BI. PE (*β* = 0.21), FC (*β* = 0.25), PT (*β* = 0.25), and SE (*β* = 0.19) were significant factors influencing the intention to use telerehabilitation (*p* < 0.05). There was no multicollinearity found between the constructs because the variance inflation factor (VIF) values were all ⩽ 3 (22).

## Discussion

This study investigated the BI and potential factors influencing the use of telerehabilitation by people with rehabilitation needs. Overall, the individuals’ use intention telerehabilitation is high. Our results indicated that individuals with severe self-assessed health conditions have higher use intentions for telerehabilitation, and PE, FC, PT, and SE are important factors influencing the intention to adopt telerehabilitation for individuals with rehabilitation needs.

Inconsistent with our findings, some previous studies indicated that the other user group (e.g., diabetic, chronic pain, and hospitalized) acceptance of telemedicine was only low-moderate (21, 23, 24). This may be because these studies were done 7 years ago, during which time telemedicine has gradually entered the public’s life with the rapid development of communication technology and the popularization of smart devices and has received increasing attention from a growing number of patients. On the other hand, the COVID-19 pandemic has hindered the traditional face-to-face medical service model, which in turn has promoted the development of telemedicine.

Our study findings show that the sociodemographic characteristics (e.g., age, gender, education, and occupation) had no significant effect on BI, which is similar to the findings of Yousef et al. (25) and Bäuerle et al. (26). However, it is worth noting that the findings indicated that individuals with self-assessed health conditions that severely impacted their lives had a higher adoption intention for telerehabilitation than individuals who were not affected. This may be because individuals with severe self-assessed health conditions have a stronger motivation to rehabilitate, and they are more eager to try this new and effective form of rehabilitation medical service. Meanwhile, individuals with rehabilitation needs are often required to have long-term and continuous rehabilitation medical services, whereas the traditional face-to-face rehabilitation medical services require them to travel frequently between their residence and the hospital, so they are more willing to adopt the convenient telerehabilitation.

The results of the study indicated that the main constructs of UTAUT, PE, and FC significantly influenced the public’s intention to adopt telerehabilitation. Some previous studies that have applied the UTAUT model to identify the important factors influencing the use of telemedicine have also established similar results (27–29). The findings suggested that the individuals’ foremost focus when using telerehabilitation remains on whether telerehabilitation can meet their actual rehabilitation needs. Telerehabilitation has the ability to provide tailored interventions to their needs and preferences, which is important for individuals with rehabilitation needs. FC was categorized into internal and external factors. External factors depend mainly on network conditions, device support, and so on. Internal factors include timely technical support and assistance (14). A key characteristic of telemedicine was the need for an infrastructure to match it. A previous study showed that the recipients of telemedicine were concentrated in urban areas with better infrastructure, while 91–99% of rural areas did not have telemedicine (30). Population groups who have higher needs for healthcare services and have the potential to benefit most from telemedicine are the ones who will encounter the greatest barriers to accessing telemedicine services (31). In other words, the essence of telemedicine is the application of advanced communication technologies in the medical field, so individuals first need to have the infrastructure and technical support of these advanced telecommunication technologies in order to access telerehabilitation rehabilitation medical services. However, our study found that EE and SI had no significant effect on adoption intention for telerehabilitation, which is inconsistent with the findings of some previous studies (21, 32, 33). This may be because with the prolonged use of telecommunication technologies and the improving smart mobile devices and technologies for people, users have become proficient in using applications related to telecommunication technology. This makes the issue of ease of use of telerehabilitation services less problematic. Our opinion gained support from Yuan et al. (34), who stated that the improvements in the ease of use of smartphone interfaces have reduced the difficulties that citizens may encounter when using telemedicine services. In addition, only 19.3% of the surveys in this sample had telerehabilitation experience; in other words, telerehabilitation is not widely used in the surveyed area. Therefore, individuals may not be able to obtain a proper understanding of telerehabilitation from important people around them (family, friends, medical workers, etc.). This may be the reason why SI is not significant in the adoption intention for telerehabilitation.

In addition, our findings revealed that PT and SE had a significant positive effect on the adoption intention for telerehabilitation. This is consistent with the results of Zhu et al. (14) and Mensah et al. (35). Telemedicine is closely related to an individual’s health; when individuals are using the process of telerehabilitation, they should be provided with accurate and reliable professional telerehabilitation medical services to enhance their trust in telerehabilitation and thus promoting the user’s intention to adopt. Absolutely, SE also plays an important role in the adoption of telerehabilitation as individuals seek to access and enjoy quality telerehabilitation medical services.

## Limitations

There are several limitations of this study. First, this study is a single-center survey research, and the sample size was not very large, so there may be a sample bias. In addition, only 19.3% of the participants in this survey had experience in the use of telerehabilitation, and most of them may not have a good understanding of telerehabilitation, which may affect the accuracy of the results in this case. Finally, this study only investigated the use intention of telerehabilitation but not the actual usage behavior. Although use intention is a predictor of actual usage, there is an “intention-behavior gap” (36). Therefore, the actual use of telerehabilitation needs to be further examined in future studies.

## Conclusion

This study investigated the use intention of telerehabilitation for individuals with rehabilitation needs and potential influencing factors. Our findings indicate that individuals’ overall use intentions for telerehabilitation are high. Health conditions, PE, FC, PT, and SE are important factors influencing the intention to adopt telerehabilitation for individuals with rehabilitation needs. Telerehabilitation is a new model of rehabilitation medicine that can provide long-term and professional rehabilitation medical services for individuals with rehabilitation needs. When promoting the use of telerehabilitation in clinical settings, relevant clinicians or healthcare organizations need to consider the important influencing factors observed. In future, large-scale investigations are still needed to gain a comprehensive understanding of the influencing factors of the intention to use telerehabilitation.

## Data availability statement

The raw data supporting the conclusions of this article will be made available by the authors, without undue reservation.

## Ethics statement

The studies involving humans were approved by the Ethics Committee of the First Affiliated Hospital of Gannan Medical University. The studies were conducted in accordance with the local legislation and institutional requirements. The participants provided their written informed consent to participate in this study.

## Author contributions

M-YW: Writing – review & editing. HC: Data curation, Methodology, Writing – original draft. CG: Investigation, Project administration, Writing – original draft. X-MP: Writing – original draft. Y-BZ: Writing – original draft. C-MW: Supervision, Writing – review & editing. YL: Writing – review & editing. Y-QW: Data curation, Investigation, Methodology, Writing – original draft.
