# Supplementary material for: Understanding the use intention and influencing factors of telerehabilitation in people with rehabilitation needs: a cross-sectional survey
Source: Front Public Health. 2023 Oct 31;11:1274080. doi: 10.3389/fpubh.2023.1274080 (PMC10654628; doi:10.3389/fpubh.2023.1274080)
Supplement: Supplementary file 1 [file Data_Sheet_1.DOCX]

Appendix 1

Table 1 Measurement items of the constructs.

| Construct | Item | Questions | Source |
| --- | --- | --- | --- |
| Performance Expectancy (PE) | PE1 | I think telerehabilitation can help me with my child's home rehabilitation | Deng Z et al. (2018)[1]  Zhang Y et al. (2019)[2]  Serrano KM et al. (2020)[3] |
|  | PE2 | Telerehabilitation providers can provide individualized rehabilitation services for children based on the home environment and individual needs |  |
|  | PE3 | Telerehabilitation can relieve my anxiety when performing home rehabilitation for my children |  |
|  | PE4 | Telerehabilitation helps children with on-going home rehabilitation |  |
| Effort expectancy (EE) | EE1 | I think I can easily use intelligent devices related to telerehabilitation (e.g., smartphones, tablets, etc.) | Deng Z et al. (2018)[1]  Zhang Y et al. (2019)[2]  Serrano KM et al. (2020)[3] |
|  | EE2 | I think my communication and interaction with the telerehabilitation provider is easy |  |
|  | EE3 | The operation process of telerehabilitation is simple |  |
| Social influence (SI) | SI1 | Doctor's recommendation and advocacy will encourage me to use telerehabilitation | Deng Z et al. (2018)[1]  Zhang Y et al. (2019)[2]  Serrano KM et al. (2020)[3] |
|  | SI2 | Family support will motivate me to use telerehabilitation |  |
|  | SI3 | The use of other patients and their caregivers will prompt me to use telerehabilitation |  |
| Facilitating conditions (FC) | FC1 | I have the resources necessary to use telerehabilitation (e.g., smartphone, tablet, computer, traffic, etc.) | Deng Z et al. (2018)[1]  Zhang Y et al. (2019)[2]  Serrano KM et al. (2020)[3] |
|  | FC2 | I have the knowledge necessary to use telerehabilitation |  |
|  | FC3 | When using telerehabilitation, I can make an appointment with a telerehabilitation provider for telerehabilitation based on my schedule |  |
|  | FC4 | The home environment (the space needed for home rehabilitation training, teaching aids, etc.) can support me in using telerehabilitation |  |
| Perceived risk (PR) | PR1 | I am concerned that the hospital and associated staff will reveal my personal privacy when I use remote rehabilitation | Deng Z et al. (2018)[1]  Cao J et al. (2022)[4] |
|  | PR2 | Uncontrollable training accidents may occur when using telerehabilitation |  |
|  | PR3 | When using telerehabilitation, there may be elements of the rehabilitation instruction that I cannot understand |  |
|  | PR4 | When using telerehabilitation, the child's compliance with the rehabilitation is poor, which may increase my psychological burden |  |
| Trust | Trust1 | The telerehabilitation provider is a professional recommended by the hospital and is a professional and authoritative | Deng Z et al. (2018)[1]  Lee WI et al. (2021)[5] |
|  | Trust2 | I trust the professionals who provide me with rehabilitation guidance |  |
|  | Trust3 | I think the telerehabilitation is trustworthy |  |
| Behavioral intention (BI) | AI1 | I would like to continue using telerehabilitation | Deng Z et al. (2018)[1]  Zhang Y et al. (2019)[2]  Serrano KM et al. (2020)[3] |
|  | AI2 | I will recommend it to those around me who need it |  |
|  | AI3 | I believe that many people will choose to use telerehabilitation in the future |  |
